# The Influence of Sex on the Slaughter Parameters and Selected Blood Indices of Greenleg Partridge, Polish Native Breed of Hens

**DOI:** 10.3390/ani11020517

**Published:** 2021-02-17

**Authors:** Kornel Kasperek, Kamil Drabik, Katarzyna Miachalak, Dorota Pietras-Ożga, Stanisław Winiarczyk, Grzegorz Zięba, Justyna Batkowska

**Affiliations:** 1 Institute of Biological Basis of Animal Production, University of Life Sciences in Lublin, 13 Akademicka St., 20-950 Lublin, Poland; kornel.kasperek@up.lublin.pl (K.K.); kamil.drabik@up.lublin.pl (K.D.); grzegorz.zieba@up.lublin.pl (G.Z.); 2 Department of Epizootiology and Clinic of Infectious Diseases, University of Life Sciences of Lublin, 30 Głęboka St., 20-612 Lublin, Poland; epizootiologia@gmail.com (K.M.); dorota.ozga@up.lublin.pl (D.P.-O.); stanislaw.winiarczyk@up.lublin.pl (S.W.)

**Keywords:** local breed, capons, conservative stock, serum parameters

## Abstract

**Simple Summary:**

For the last decade, capons have become very popular because of the specific quality traits of their meat. In case of laying hens caponization may be a solution to the problem of redundant roosters. The Greenleg partridge breed, covered by a program for livestock genetic resources conservation in Poland, consisted of attraction, as the oldest Polish hens breed, as well as the source of poultry products (eggs and meat) in a small farm. The cockerels are often intended for caponization, due to Polish tradition and consumer preferences. However, this procedure influences not only their productivity or meat quality, but also physiological aspect of birds. We were interested not only in capons compared to roosters, but also in relation between sexes in case of productive traits as well as their selected physiological traits. The sex effect was found in all experimental aspects. Cocks had the highest carcass yield, however, the biggest breast muscles proportion were stated in capons carcasses. The highest proportion of abdominal fat pad was found in females but the lack of sex hormones in capons also contributed to a higher fat accumulation. The serum profile showed that the sexual maturity of hens increased lipids content (cholesterol, trigliceroles) caused by laying production.

**Abstract:**

The aim of the study was to assess the influence of sex, including caponization, on selected physiological and productive traits of Greenleg partridge (GP) birds. The study material consisted of 120 GP chicks (40 females and 80 males), divided into 3 equal groups (4 replication in each) and kept in litter system and fed a*d libitum*. A total of 40 cocks have been surgically castrated. The body weight (BW) of birds were measured biweekly. At the age of 24 weeks 8 birds/group were slaughtered, their carcasses were subjected to simplified dissection. Blood samples were collected and among others biochemical profile of serum was established. The lowest BW, regardless of age, had hens. From 18th week capons had the highest BW and finally it was similar to cocks. Cocks demonstrated, significantly, the highest carcass yield, however, the biggest proportion of breast muscles were stated in capons carcasses. The effect of sex is very clear in case of abdominal fat pad. The highest proportion of it was found in females but the lack of sex hormones in capons also contributed to a higher fat accumulation. The serum profile showed that the sexual maturity of hens increased lipids content (cholesterol, trigliceroles) caused by laying production.

## 1. Introduction

Although nowadays most poultry production is based on selected high-productive hybrids, more and more attention is being paid to the need to preserve native breeds. In Poland, 19 lines of laying hens [1] are currently under protective status. One of them is the Greenleg Partridge (GP), which is one of the oldest and only non-hybrid hen breeds kept in Poland. The GP was separated as a breed at the end of the 19th century from the so-called “Galician hens” with green shank color and partridge plumage. The first time these hens were exhibited under the name of the Greenleg in 1894 at the General National Exhibition in Lviv (now in Ukraine). This breed, originally occurs in south-eastern Poland after World War II, was subjected to regionalization to this area. At this time, two Greenleg Partridge stocks are kept, the GP strain with about 1000 hens and 200 roosters at the Laura Kaufman Didactic and Research Station of Small Animals that belongs to the University of Life Sciences in Lublin, and the second population of these birds (Z-11) at the Experimental Department of the Institute of Zootechnics, National Research Institute in Chorzelów. GP strain analyzed in the study is considered to be the oldest and most primitive genotype of these birds, maintained without foreign blood supply since 1945.

Greenleg Partridge were the basis for the creation of the only autosexing Polish hen breed which is Polbar derived from the crossbreeding of Barred Plymouth Rock and GP during multi-generational breeding work [2]. The only Polbar hen population in the world is maintained as a conservative stock belonging to the University of Life Sciences in Lublin (Poland) [3].

Currently, the most important role of such breeds as Greenleg Partridge is to preserve genetic biodiversity. The strategy of including old local breeds in the ex-situ conservation program allows for the preservation of a valuable gene pool [4] and biologically important features of birds. Research is still conducted on molecular genetics [5], physiology [6,7], stress resistance [8], behavior [9], and production characteristics. GPs, due to their resistance to diseases, food foraging prowess, strong maternal instinct, intensive broodiness, as well as adaptability to environmental conditions has often been the material for hybrids of both, laying [10] and meat utility type [11], dedicated to extensive (i.e., organic) systems of rearing. Recently, these birds have gained popularity, due to the consumers’ conviction about the reduced cholesterol content in the obtained eggs. The results of research in this area are not entirely clear, due to the fact that some authors indicate the existence of such a relationship [12], while others do not confirm similar observations [13].

The intensification of laying poultry production, in which the Greenleg Partridge is classified, is associated with an increase in the number of unnecessary cockerels, creating a problem for farms to manage them. It also happens that with a relatively small production of chicks, as is the case with breeding flocks of conservative breeds, it is not possible to separate them according to their sex and it is necessary to rear birds of both sexes until they start to show the sexual dimorphism based on morphological characteristics.

Not only in Poland, but also in the world, one of the methods of using roosters is their caponization. Maintaining capons is also a response to an ethical problem related to the killing of day-old chicks, which raises considerable controversy in society. The procedure of male birds castration has been known since ancient times, where it was performed to obtain heavy birds with good meat quality. At present, a wide range of countries such as China and the United States [14] are carrying out cock castration on a large scale. Importantly, some countries also see caponization as an opportunity for native breeds [15], because slow-growing birds are most frequently used [1].

In the case of caponization, most of the works use cocks and capons of a specific type (breed, hybrid) of research material. However, it seems that in the context of conservative breeds, they should be treated comprehensively, especially if they are intended to constitute a source of poultry material in extensive farms. Therefore, females should also be considered in addition to males. There are several articles in the available literature on the quality of meat of Greenleg Partridge capons compared to cocks [16,17], but there are no similar reports concerning hens of this breed. At the same time capons may be treated as the 3rd “sex category” which may influence not only the production effects or quality of meat but also some physiological traits of birds. The choice of Greenleg partridge was motivated by the fact that these purebred hens were kept for many generations without any genetic improvement (selection) what may allow to obtain more reliable results.

The aim of the study was to assess the influence of sex, including the caponization procedure, on selected physiological and productive features of Greenleg Partridge birds.

## 2. Materials and Methods

The research was conducted with the approval of the Local Ethical Committee (No. 101/2017), Lublin, Poland.

The study material consisted of Greenleg partridge chicks. Birds derived from the conservative stock at Laura Kaufman Didactic and Research Station of Small Animals that belongs to the University of Life Sciences in Lublin, Poland. Typically, day-old-birds are sexed and intended for the stock renovation in ratio 1♂:8♀. Up to the age of 6 weeks birds of both sexes were reared together and then sexed. 

In total, 120 birds (40 females and 80 males) were separated by sex based on their morphological characteristics (plumage, comb size) and then randomly divided into 3 equal groups (40 birds in each). Each group was additionally divided into replication subgroups (á 10 chicks) and kept in litter pens with constant access to water and feed (*ad libitum*), maintaining the lighting program standard for rearing laying hens. The feed mixture was adjusted to the age and physiological condition of the birds and its basic chemical composition determined using feed analyzer NIRS™ DA1650(FOSS Co., Hilleroed, Denmark), is presented in Table 1. 

A total of 40 cockerels (6 weeks old) have been surgically castrated by an authorized veterinarian. The surgical procedure was performed under general anesthesia (sub-dissociative ketamine 10%) intramuscularly 0.2 mL/individual. Before surgery, birds were subjected to a 12-h feed withdrawal with access to water. To perform the procedure, 2 incisions were made on both sides of the body in the line of the hip joint, from the top about 1−2 cm from the transverse processes towards the edge of the last rib. After inserting the dilator and dissecting the air sac the testicles were cut off by gently placing a loop over them. The procedure was performed on poorly bloodied tissues; therefore, there was no need to stop bleeding. The wound was closed immediately after surgery by the abdominal integuments sliding over each other under the influence of their elasticity.

At 14 day intervals, birds were weighed to determine weight gain. At the age of 24 weeks, 2 birds were randomly selected from each replication subgroup (8 from per group) and slaughtered in commercial poultry abattoir by decapitation following electrical stunning using an electric current of 45 mA. Birds were subjected to feed restriction 8 h before slaughter. The procedure of slaughtering was in accordance with EU Regulation No. 1099/2009 of 24 September 2009 on the protection of animals at the time of slaughtering. 

After plucking and evisceration, the carcasses were chilled by air method (0 °C, 4 h) and subjected to dissection analysis. Following parts of carcasses were extracted during the dissection [18]: breast muscles, thighs, drumsticks, wings, and trunk. Edible giblets (heart, liver, gizzard) were weighed to assess their proportions in body weight. Hot carcass well as its part were also weighted and their percentage share was calculated (as the proportion of the weight of particular carcass parts to its total weight). 

During the slaughter blood was taken to test tubes containing EDTA (ethylenediaminetetraacetic acid) as anticoagulant and to tubes not containing anticoagulant for biochemical analyses. All samples were analyzed triple. The hematocrit (HT, packed cell volume) was determined by the microhematocrit method in capillary tubes and centrifuged at 10,000 RPM for 3 min. Heamaglobin content was calculated by dividing the hematocrit value by 3. 

A smears was made of the whole blood, which was fixed and stained with the use of a commercial Hemacolor^®^ dye set (Sigma-Aldrich Co., Ontario, Canada), following the procedures recommended by the manufacturer. Then the prepared preparations were observed with DELTA^®^Optical Microscope (Delta Optical, Nowe Osiny, Poland) at magnification ×1000 (eyepiece ×10, lens × 100) using immersion oil. The number of heterophilia and lymphocytes was observed by taking morphological markers of blood cells according to those proposed by Lucas [19]. 

After collection, the blood, taken to tubes without anticoagulant, was incubated at 37 °C until a natural clot was formed. Then the blood plasma was taken to 2 mL eppendorff tubes for biochemical tests. Plasma samples were analyzed using BS 130 Mindray apparatus (Shenzhen Mindray Bio-medical Electronics Co., China) to determine cholesterol (CHOL), alanine transaminase (ALT), aspartate transaminase (AST), urea acid (UREA), enzymatic creatinine (CREA), trigliceroles (TG), amylase (AMYL), lipase (LIPA). Commercial analytical kits were used (Biomaxima^®^, Lublin, Poland).

The data were analyzed with the use of the statistical package SPSS 24.0PL [20]. The Kolmogorov–Smirnov test was carried out in case of normality of data. The significance level was defined as 5%. The obtained numerical data were verified by one-factorial ANOVA and Tukey’s test.

## 3. Results

As a result of surgical procedure directly after it, the mortality of 4 birds was registered (6.6%). Later, during the rest of the rearing period any mortality incidences were noticed.

**Figure 1:**
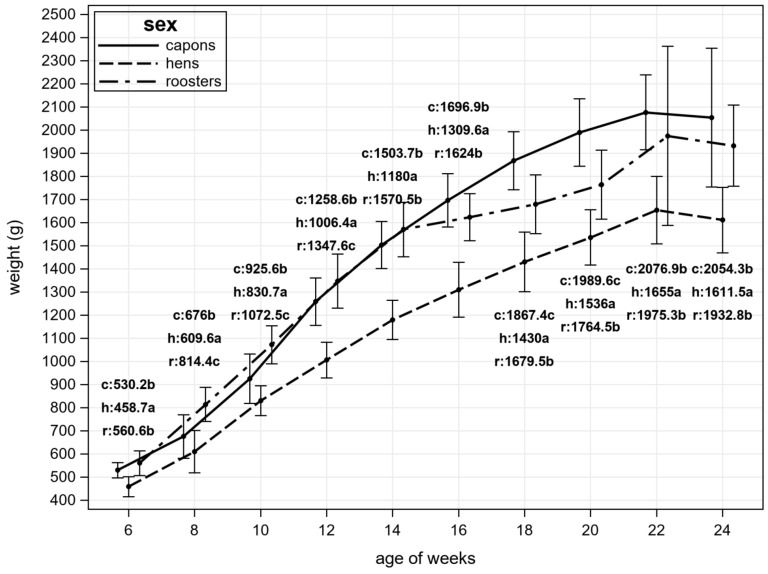
The body weight of roosters (r), hens (h), and capons (c) depending on their age; a,b,c means differ significantly at *p* ≤ 0.05 (Tukey test).

From the moment the birds were selected for the experiment, their body weight was controlled and the results are presented in Figure 1. It should be noted that the birds were weighed for the first time after they were properly divided into experimental groups, so the lower body weight of capons compared to roosters is not caused by differences in the initial weight of birds and the caponization procedure itself and the period of bird convalescence. The lowest body weight, regardless of the date of measurement, was characteristic for hens. In the case of capons and roosters, it was observed that up to 14 weeks of birds’ life the capons were characterized by significantly lower body weight. At 16 weeks there were no significant differences in body weight between castrated and uncastrated males. The capons had the highest body weight of all experimental groups at 18 and 20 weeks of age.

The results of the slaughter analysis of birds included in the experiment are presented in Table 2 and Table 3. In birds subjected to the slaughter analysis it was found that the hens had the lowest body weight, with the highest value of this trait for capons. It was observed that significantly the highest proportion of heart mass was found in cocks, with no differences between hens and capons. In case of the liver proportion in the body weight of birds, it was observed that males, regardless of the surgical castration, did not differ significantly and was considerably lower compared to the liver weight of the hens included in the experiment.

The biggest differences were observed in the proportion of abdominal fat pad in the birds’ body weight. The highest values were found in hens, while in cocks the value was significantly the lowest and almost four times smaller than in hens, and more than twice as that observed in capons.

The highest carcass yield were found in cocks, with the lowest value recorded for hens (Table 3). At the same time, it should be noted that in the case of the most valuable carcass part, the breast muscle, an inverse relationship has been observed. At the same time, it should be noted that both in the case of carcass yield and the proportion of breast muscles in the carcass weight, the capons did not differ significantly from hens and roosters. 

The proportion of thighs in carcasses was similar in all groups regardless of the sex of birds, while significant differences were observed in the drumsticks weight. Hens and capons were characterized by a significantly lower percentage of this element in comparison to cocks. In the case of the rest of carcass elements, there were no significant differences in their percentage share, regardless of the sex of birds.

The results of morphological and biochemical analyses of birds’ blood depending on their sex are presented in Table 4. Hematocrit (HT) analysis showed that the most morphotic elements contained blood of cocks, significantly the least hens. Similar observations concern the level of haemoglobin in birds’ blood, whose highest values were recorded for Greenleg roosters with no discrepancy between hens and capons. Interestingly, the influence of the sex of birds on the heterophyll–lymphocyte ratio, which is an indirect marker of bird welfare, was not demonstrated.

No significant differences were found for AST, ALT, UREA, Crea, and LIPA. However, significant differences were observed for amylase activity. The highest values of this parameter characterized the capons, with the lowest values recorded in hens. At the same time, it is worth noting that all groups differed significantly in the activity of this enzyme.

## 4. Discussion

The growth rate of birds depends on their sex. Similar observations apply to the Greenleg Partridge. Reports on the various breeds cocks caponization impact on the body weight gain are not consistent. Some authors have shown that this treatment contributes to the increase in birds’ body weight [16], while others did not show similar relationship [21]. The results of own research are similar to those carried out by Zawadzka et al. [22], although in contrast to the research of the indicated authors, the results of own research suggest that GP capons gained a higher weight compared to cocks only 8 weeks after the castration.

In the case of GP hens, there is no data on their growth rate. The differences observed in the study are due to sexual dimorphism in hens, which makes males, depending on the breed, even more than 20% heavier than females [23]. At the same time, it should be noted that the results obtained from own research do not differ from the results of the observations carried out for the conservation of genetic resources program [24].

The results of our own research, additionally, indicate two characteristic “peaks” in body weight gain. In the case of roosters (14 weeks) it could be closely related to the sexual maturity achievement of males, which is confirmed by research conducted by Zawadzka et al. [22], which observed a significant increase in testosterone levels in GP roosters after 12 weeks of age. At that time, in spite of increased vocalization, the birds were also establishing a hierarchy in their stocks through aversive behavior, which influenced their production result. Additionally, in the case of hens a decrease in growth rate can be observed. As in the case of cocks, it was caused by physiological changes in the birds’ body. The growth curve is flattened when the birds start the laying production [25] which correlates with our own observation.

The weight of organs varies considerably according to the sex of birds. Many works indicate a significantly higher liver mass recorded in capons compared to cocks [26,27]. In our study, no similar relation was observed, neither was in the study of Zawadzka et al. [22].

In terms of heart mass, some authors [16,28] do not find significant differences, while others report that the heart mass of caponized birds was significantly higher than that of cocks [29]. In our study, the opposite relationship was found. Probably this effect is related to the higher activity of birds or their behavior. This is partly confirmed by studies of Cunningham et al. [30], who showed that heart size was related to the hen’s position in the stock, the lower-ranking birds had significantly larger hearts. As has already been mentioned for the body weight of birds, cocks showed behavior indicating attempts to establish a stock hierarchy. Therefore, this relationship may affect the significantly highest heart weight of cocks recorded in our study.

In the case of hens it was observed that the proportion of heart in body weight was significantly lower compared to that recorded for cocks. Similar conclusions were also obtained by Tasoniero et al. [31], who demonstrated the important role of sex in heart weight formation in two Italian local breeds. Interestingly, these observations do not correspond to those reported by Musundire et al. [32], who did not demonstrate a significant influence of bird sex on heart size.

Higher, compared to cock carcasses, fattening of capons has already been described by many authors [33,34], what is consistent with our own observations. A higher degree of fat deposition is often associated with a decrease in testosterone levels, which is the result of testicular removal during surgical caponization [35].In the case of hens, research carried out on broilers showed that hens had a higher fat content [36], which is consistent with the results of our study. Unfortunately, in the case of laying hens, there are no similar reports, as most authors focus on reducing abdominal fat in laying hens by dietary supplementation, i.e., using wild ginseng or dihydropyridine [37,38], because it has been shown that an excessive amount of abdominal fat results in a reduction in reproductive abilities [39].

The available literature data indicate higher slaughter performance of males compared to females [36,40], which is also confirmed by the results of our findings. On the other hand, in case of capons, lower carcass yield is confirmed in many works [16,41]. This relationship is closely related to the content of abdominal fat, which is removed together with the gizzard and intestines during the evisceration of birds.

At the same time, according to Tor et al. [41], the lower weight of the capon carcass is compensated for by its better muscularity. It is partially confirmed by the higher proportion of breast muscles in the capon carcasses compared to other groups. A higher proportion of pectoral muscle in the capons was also observed by Miguel et al. [42] and Kwiecień et al. [16]. However, Volk et al. [43] did not demonstrate the role of the caponisation in pectoral muscle mass. Rizzi et al. [44] pointed to a similar share of pectoral muscle in hens belonging to local Italian breeds. It should be also noticed that the difference observed indicates a relationship between muscle content and sex of birds, which is in line with the data presented by Cassandro et al. [45], who showed an significant impact of the interaction between age and sex of birds on breast muscle traits including its mass.

Haematocrit, as well as the amount of haemoglobin associated with it, are related to the sex of birds [46] which is also confirmed by our research. In case of capons, it was found that both hematocrit and haemoglobin values are significantly lower in them than in uncastrated cocks. These observations are also confirmed by studies of other authors [26,47].

However, the greatest differences were observed in the level of cholesterol and triglyceride. Male castration contributes to an increase in triglyceride and cholesterol levels, as confirmed by studies conducted by Guo et al. [48] or Muhmad et al. [47]. Similarly to abdominal fat, the difference in testosterone levels constitutes the basis for this variability. In the case of hens, significantly the highest cholesterol and triglyceride scores were recorded, but this variability is mainly caused by physiological factors [49]. Hawkins and Heald [50] in their work clearly indicated that the level of total lipids concentration in the blood of sexually immature birds is significantly lower than that of hens entering the laying production. These studies somehow confirmed the results of our research, as at the age of 22 weeks GP hens start the eggs production.

Taking into account the fact that birds were fed with exactly the same balanced feed mixture and kept under the identical conditions, the results on plasma amylase levels indicate a dependence on the sex of the birds. Interestingly, the amylase level is related to bird stress, or rather to the level of corticosterone [51]. However, analysis of other blood parameters (ALT, AST, H:L) seems to deny the possibility of additional stress in only one sex (group) of birds. It is therefore necessary to carry out additional studies that may explain the birds’ sex influence on their plasma amylase level.

## 5. Conclusions

The sex, as well the caponization effect was found in all experimental aspects, from productivity to selected physiology traits of birds.

The lowest BW, regardless of age, had hens. From 18th week capons had the highest BW and finally it was similar to cocks. Cocks had the highest carcass yield, however, the biggest breast muscles proportion were stated in capons carcasses.

The highest proportion of abdominal fat pad was found in females but the lack of sex hormones in capons also contributed to a higher fat accumulation. The serum profile showed that the sexual maturity and laying production of hens increased lipids content (cholesterol, trigliceroles) what may result from their subsequent deposition in the egg yolk.

Despite standardized rearing conditions, age and breed of birds, significant differences were observed in almost all parameters included in the study. This clearly indicates the influence of sex on production traits and blood biochemical parameters. It should be noted at the same time that the results obtained for capons were between those observed for hens and roosters. This suggests the influence of sex hormones, but additional studies are necessary to provide evidence of this relationship especially in case of hens biochemical parameters.

## Figures and Tables

**Table 1:** Basic chemical composition of feed mixtures.

Ingredients	1–8 Weeks Old	8–18 Weeks Old	>18 Weeks Old
ME (MJ kg^−1^)	11.20	11.30	11.00
Crude protein (%)	17.32	15.24	16.72
Crudefat (%)	4.00	4.00	3.40
Crudefibre (%)	5.50	6.20	7.15
Crudeash (%)	4.45	4.52	5.00

**Table 2:** The slaughter body weight and giblets proportions of birds depending on their sex.

Trait		Rooster		Hen		Capon		*p*-Value
		Mean	SD	Mean	SD	Mean	SD	
body weight (g)		1911.9 ^b^	85.02	1582.1 ^a^	48.57	2050.4 ^c^	81.03	0.000
giblets (%)	heart	0.499 ^b^	0.04	0.338 ^a^	0.06	0.349 ^a^	0.05	0.000
	liver	1.257 ^a^	0.13	1.552 ^b^	0.27	1.284 ^a^	0.07	0.006
	gizzard	1.224	0.20	1.509	0.38	1.288	0.28	0.155
abdominal fat pad (%)		0.89 ^a^	0.17	4.14 ^c^	2.23	2.77 ^b^	0.44	0.000

^a, b, c^ means within row differ significantly at *p* ≤ 0.05 (Tukey test).

**Table 3:** The carcass parameters of birds depending on their sex.

Trait		Rooster		Hen		Capon		*p*-Value
		Mean	SD	Mean	SD	Mean	SD	
carcass yield (%)		71.13 ^b^	1.39	65.27 ^a^	3.76	68.08 ^ab^	1.52	0.000
carcass cuts (%)	breast muscles	19.16 ^a^	1.07	21.2 7 ^b^	2.50	20.09 ^ab^	0.40	0.046
	thights	18.82	1.35	16.88	2.17	17.23	1.06	0.054
	drumsticks	15.23 ^b^	1.43	12.84 ^a^	1.41	13.41 ^a^	0.94	0.003
	wings	11.94	0.67	12.02	0.96	11.96	0.38	0.967
	trunk	34.86	1.90	36.98	6.81	37.31	1.71	0.460

^a, b^ means within row differ significantly at *p* ≤ 0.05 (Tukey test).

**Table 4:** The blood and serum indices of birds depending on their sex.

Trait	Rooster		Hen		Capon		*p*-Value
	Mean	SD	Mean	SD	Mean	SD	
HT (%)	41.81 ^b^	3.53	30.24 ^a^	2.96	32.94 ^a^	2.58	0.000
HGB (g/dL)	13.95 ^b^	1.18	10.09 ^a^	0.98	10.99 ^a^	0.86	0.000
H:L	0.34	0.10	0.32	0.08	0.35	0.20	0.913
Chol (mg/dL)	146.13 ^a^	5.00	266.50 ^b^	82.41	155.00 ^a^	8.26	0.000
ALT (u/L)	43.13	107.24	25.96	21.80	5.38	4.63	0.501
AST (u/L)	256.50	108.43	268.88	52.36	272.38	36.56	0.900
UREA (mg/dL)	25.75	1.67	48.50	70.52	23.50	1.07	0.413
Crea (mg/dL)	0.31	0.02	0.30	0.03	0.29	0.04	0.652
TG (mg/dL)	76.50 ^a^	25.23	1197.50 ^b^	103.40	82.25 ^a^	23.09	0.000
AMYL (u/L)	648.25 ^b^	95.63	406.75 ^a^	57.81	819.00 ^c^	139.67	0.000
LIPA (u/L)	94.61	104.94	90.89	87.30	37.95	69.87	0.376

HT – hematocrit; HGB – heamaglobin content; H:L – heterophyll–lymphocyte ratio; Chol – cholesterol; ALT – alanine transaminase; AST – aspartate transaminase; UREA – urea acid; Crea – enzymatic creatinine; TG – trigliceroles; AMYL – amylase; LIPA – lipase; ^a,b,c^ means within row differ significantly at *p* ≤ 0.05 (Tukey test).

## Data Availability

Not applicable.
